# The superoxide scavenger TEMPOL induces urokinase receptor (uPAR) expression in human prostate cancer cells

**DOI:** 10.1186/1476-4598-5-21

**Published:** 2006-06-06

**Authors:** Danielle Lejeune, Mohammad Hasanuzzaman, Amanda Pitcock, Joseph Francis, Inder Sehgal

**Affiliations:** 1 LSU Department of Comparative Biomedical Sciences, Louisiana State University, Baton Rouge, LA, 70803, USA

## Abstract

There is little understanding of the effect that reactive oxygen metabolites have on cellular behavior during the processes of invasion and metastasis. These oxygen metabolites could interact with a number of targets modulating their function such as enzymes involved in basement membrane dissolution, adhesion molecules involved in motility or receptors involved in proliferation. We investigated the effect of increased scavenging of superoxide anions on the expression of the urokinase receptor (uPAR) in PC-3M human prostate cancer cells. Urokinase receptor is a GPI-linked cell surface molecule which mediates multiple functions including adhesion, proliferation and pericellular proteolysis. Addition of the superoxide scavenger 4-hydroxy-2,2,6,6-tetramethylpiperidinyloxy (TEMPOL) to PC-3M cultures stimulated expression of uPAR protein peaking between 48 and 72 hours. Cell surface expression of the uPAR was also increased. Surprisingly, uPAR transcript levels increased only slightly and this mild increase did not coincide with the striking degree of protein increase. This disparity indicates that the TEMPOL effect on uPAR occurs through a post-transcriptional mechanism. TEMPOL presence in PC-3M cultures reduced intracellular superoxide-type species by 75% as assayed by NBT dye conversion; however this reduction significantly diminished within hours following TEMPOL removal. The time gap between TEMPOL treatment and peak uPAR protein expression suggests that reduction of reactive oxygen metabolites in prostate cancer cells initiates a multistep pathway which requires several hours to culminate in uPAR induction. These findings reveal a novel pathway for uPAR regulation involving reactive oxygens such as superoxide anion.

## Findings

Reactive oxygen species (ROS) are becoming increasingly associated with several aspects of cancer progression including not only carcinogenesis but also tumor cell proliferation and invasion [1]. In prostate cancer, oxygen radicals are reported to arise from several sources within the cells including the NADPH oxidase [1], mitochondrial glycerophosphate-dependent ROS [2], xanthine oxidase and nitric oxide synthases [3]. The cell's net redox state is a balance between oxygen radical synthesis and breakdown, and net ROS metabolism in prostate cancer arises via activities of the scavenger enzyme systems catalase, superoxide dismutase I (Zn2+/Cu2+ SOD) and II (MN-SOD), and glutathione peroxidase [3].

It has previously been reported that increased ROS precipitates the increase of some matrix metalloproteinases [4]. In addition to the important role of these metalloproteinases, prostate cancer invasion is also highly dependent upon the activity of the urokinase proteinase system consisting of the serine protease urokinase, its cell surface receptor uPAR and inhibitors such as plasminogen activator inhibitors [5-8]. Despite the knowledge that both ROS signaling and the urokinase receptor are key mediators of prostate cancer progression, there are no reports to date which have linked the two. We investigated the effect that increased scavenging of superoxide could have on uPAR expression. The superoxide scavenger used was TEMPOL, a water soluble cell permeant piperidine nitroxide [9].

**Figure 1:**
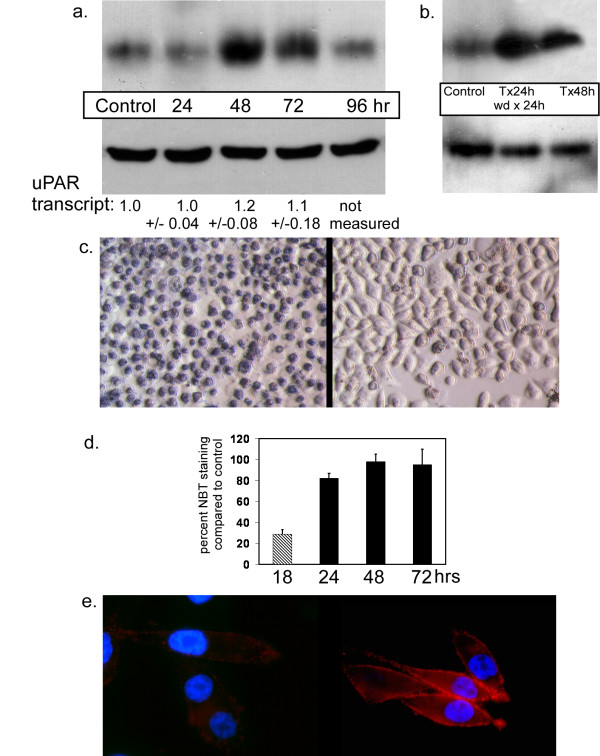
Transient TEMPOL treatment of PC-3M cells induces uPAR transcript and protein expression. (A). TEMPOL was added to cells (time 0) and then thoroughly washed away after 24 hours. Proteins were isolated at the time of TEMPOL removal (i.e. 24 h) and at 48, 72 and 96 hours and these proteins were immunoblotted for uPAR expression (upper panel). β-actin loading is shown (lower panel). uPAR transcript expression relative to control (= to 1.0) measured using real-time PCR is shown beneath each band (mean transcript ± s. dev. (B). TEMPOL was added to cell cultures for either a 24 hour period followed by withdrawal for another 24-hour period (labeled as "T × 24 h wd × 24 h"), or for a 48-hour continuous period (labeled as "T × 48 h"). In both treatments, cells were lysed and proteins were extracted after 48 hours. Control cells were not treated. Upper panel shows uPAR protein expression; lower panel shows β-actin expression to verify equivalent loading. (C). NBT staining in untreated cells (left panel) and in cells treated with TEMPOL for 18 hours (right panel). Images photographed at 320× using varel contrast. (D). TEMPOL reduction of superoxide levels is reversed following its removal. Cells were stained using NBT beginning at the time points shown in order to detect intracellular superoxide levels. Bars reflect the degree of NBT staining as a percent of control (untreated) cells. During the 18 hour time point (cross-hatched bar), NBT was present in the cell culture. (E). uPAR cell surface staining (red) in control (left panel) vs. cells 48 hours after TEMPOL treatment (right panel) as above. Nuclei are shown following staining with DAPI (blue). Image taken at 630×.

PC-3M cells were exposed to TEMPOL over a 24 hour period, then the chemical was removed. This treatment stimulated expression of uPAR protein first evident 48 hours after TEMPOL addition. This protein expression peaked between 48 and 72 hours (Figure 1A). To correlate protein levels with transcript expression, real-time PCR was used to measure uPAR PCR product. Surprisingly, only a small increase in RNA occurred and only at the 48 hour time period. The difference between the RNA levels preceding and during the time points of striking protein increase leads us to conclude that the effect of TEMPOL on uPAR protein expression is mediated through largely post-transcriptional mechanisms. To further determine if the effect of TEMPOL on uPAR expression results directly from TEMPOL exposure or rather through TEMPOL addition-then-withdrawal, we compared uPAR levels in cells treated with TEMPOL for 24 hours followed by TEMPOL removal with cells treated continuously with TEMPOL for 48 hours. These results demonstrate that continuous TEMPOL treatment also results in increased uPAR protein, although the level of uPAR increase is less than that seen after the 24 hour-treat-then-withdrawal protocol. This indicates that uPAR may be induced by the suppression of superoxide which follows TEMPOL treatment (Figure 1B).

To determine the efficacy of TEMPOL treatment and the length of the residual effect of TEMPOL following removal, we quantitated the accumulation of superoxide-type metabolites using NBT dye conversion in control and TEMPOL-treated PC-3M cells. This assay revealed that TEMPOL effectively reduced superoxide by approximately 75%; however, this effect lasted only while TEMPOL was present in the culture (Figure 1C and 1D). The inhibitory effect rapidly dissipated after TEMPOL removal. Immunostaining demonstrates that the increased uPAR protein induced by TEMPOL treatment leads to increased cell surface expression (Figure 1E).

These data suggest a novel regulatory mechanism for uPAR protein involving ROS such as superoxide anion which is pharmacologically scavenged by TEMPOL. This regulation appears to involve a significant degree of post-transcriptional regulation and results in peak protein expression after the TEMPOL effect on ROS dissipates. The time gap between TEMPOL treatment and peak uPAR protein expression leads us to hypothesize that TEMPOL acts through a multistep pathway which requires several hours to produce increased uPAR. This pathway may either be initiated during the period of TEMPOL treatment or initiated by its withdrawal. Mechanistically, TEMPOL treatment could alter the redox state of one or more target proteins upstream of uPAR protein synthesis. Although TEMPOL treatment reduces superoxide-type metabolites, it is also possible that TEMPOL withdrawal creates a rebound increase in oxygen radicals which promotes the increased uPAR. To investigate this possible rebound phenomenon, we treated PC-3M cells with TEMPOL for 48 hours and then extracted proteins. Immunoblot analysis again showed an increase in uPAR expression suggesting that the effects of TEMPOL treatment itself induces uPAR.

In addition to promoting cancer progression, uPAR maintains normal cellular attachment, proliferative signaling, proteolysis and chemotaxis [10,11]; therefore our results could indicate that modulation of uPAR expression by oxygen metabolite levels is one of many regulatory checks for maintenance of normal uPAR activity. However, because both ROS and uPAR are associated with prostate cancer progression it is tempting to speculate that regulation of uPAR by oxygen metabolites could facilitate motility, proteolysis and invasion. Oxygen metabolites have been shown to potentially regulate other proteins associated with invasion. For instance, Chiarugi et al [12] propose that ROS generated by integrin signaling inactivates several protein tyrosine phosphatases. In NIH-3T3 fibroblasts, inhibition of one of these phosphatases, low molecular weight phosphatase, then facilitates the activation of focal adhesion kinase [FAK, [12]].

FAK signaling is associated with prostate cancer metastasis [13] and therefore it is conceivable that ROS regulation of FAK could facilitate invasion. ROS have also been shown to induce uPAR in gastric cancer cells [14]; however in this study uPAR was induced by generating a net increase in oxygen radicals using H2O2 or a superoxide generator (phenazine methosulfate). In addition, gastric cancer uPAR induction by ROS was transcriptional and involved AP-1 binding to the uPAR promoter region [14].

We have assumed that the TEMPOL effect we observed occurs through its well known action as a scavenger of certain oxygen radicals particularly superoxide anion; however, we have not excluded the possibility that TEMPOL induction of uPAR results from an alternative pharmacologic reaction. Activity of enzymes such as superoxide dismutase will need to be altered in order to more specifically identify the metabolic components that influence uPAR expression in prostate cancer. To further define the pathway linking reactive oxygen metabolites and uPAR, it will be necessary to identify redox-sensitive molecular targets which can alter uPAR protein translation or half-live.

## Materials and methods

### Cell culture and treatments

PC3M cells were a gracious gift from Dr. Isiah Fidler (MD Anderson Cancer Center, Houston TX). These cells were maintained in Minimal Essential Medium with Earle's salts (MEM), supplemented with 10% fetal bovine serum, 100 units/mL penicillin, 100 ug/ml streptomycin, and 2.0 mM L-glutamine.

To scavenge superoxide, cells were treated with 4 mM final concentration of 4-hydroxy-2,2,6,6-tetramethylpiperidinyloxy, free radical (TEMPOL, purchased from Sigma Chemicals) diluted in HBSS-Ca, Mg. TEMPOL is a water soluble analogue of the spin label TEMPO and is a stable piperidine nitroxide which crosses the plasma membrane and acts as either an SOD mimetic or as a scavenger of superoxide anion [9].

### Chromogenic detection of superoxide

Nitro blue tetrazolium salt (NBT) was solubilized in 70% dimethyl formamide [15] then diluted in culture medium to a final concentration of 250 uM and added to live PC-3M cells for 6 hour periods. Cells were treated with TEMPOL over a 24 hour period after which the scavenging agent was removed. Conditions for this analysis were: 1) NBT added during cell exposure to TEMPOL – 18 hours after initial TEMPOL addition to culture media; 2). NBT added immediately after removal of TEMPOL (24 hours after TEMPOL addition); 3) NBT added 48 hours after initial TEMPOL addition; 4). NBT added 72 hours after TEMPOL addition. NBT forms a water-insoluble blue formazan when it reacts with intracellular superoxide demonstrating the degree of superoxide formation within the cell. To prepare for spectrophotometric analysis, cells were incubated for 20 minutes in a solution of 20% SDS and 50% dimethylformamide. The cells were detached from 24-well plates using a standard pencil eraser head and the cell solution was boiled for one minute to enhance solubiliztion of the formazan crystals. Cellular debris was removed by brief centrifugation and the supernatant was then analyzed at 570 nm with a reference of 650 nm.

### Immunofluorescence

Live cells were incubated overnight in growth medium with 5.0 ug/mL primary antibody (anti uPAR monoclonal, R&D Systems) to label cell surface uPAR. The antibody and medium were washed away and cells were then fixed in ice cold paraformaldehyde for 10 minutes. Paraformaldehyde was removed and replaced with a solution of phosphate-buffered saline (PBS) containing 2% goat sera for 20 minutes to block non-specific binding sites. Fluorescent-tagged secondary antibody (Alexa Fluor 594, Molecular Probes Inc) was then added in the blocking solution for one hour. Fluorescence was imaged through a Zeiss Axiovert 200 fluorescent microscope and recorded through an Olympus Q-Capture 5.1 mega pixel color digital camera.

### Gene expression analyses

To perform reverse-transcriptase PCR, total RNA was extracted from PC-3M cells using RNA Bee reagent (Tel Test, Friendswood, TX). After denaturation at 65°C for 5 min, RNA (2 μg) was added to 20 μl of RT mixture (10 μg/ml oligo(dT), 1× RT buffer, 0.5 mM each of four deoxynucleosides, 1 unit/μl RNase out, and 10 units of avian myeloblastosis virus reverse transcriptase (Invitrogen Corporation, Carlsbad, California). An RT minus control showed no visible bands. The RNA was reverse transcribed at 37°C for 60 min and 2 μl of the products were used as the template for PCR. Real-time PCR was carried out in a Real-time PCR System 7300 (Applied Biosystems) with standard temperature protocol and iTaq SYBR green supermix (Bio-Rad Corp.) in a 25 uL reaction volume. The default thermocycler program for all genes was: 3 minutes pre-incubation (95°C) followed by 40 cycles for 15 seconds at 95°C and one minute at 60°C. The last cycle was followed by melting curve analysis between 60 and 95°C. The individual primer concentrations in all PCR reactions were 200 nM. All amplifications were carried out in optically clear 96-well Gold PCR plate, Corning Inc.) with optical adhesive cover films. Accumulation of PCR product was detected by monitoring the increase in fluorescence of the reporter dye. Relative expression values were obtained by normalizing Ct values of the tested genes with Ct values of GAPDH using the 2-ΔΔCt method. The primers used for uPAR were 5'-ATCAGACATGAGCTGTGAGAGG-3' and 5'-ACTACGGCTCTGAAGTCACCAC-3'; these primers generated a 194 bp. PCR product.

Western Blot analysis for uPAR has been previously described [16].

## Abbreviations

NBT Nitro blue tetrazolium salt

uPAR urokinase receptor

TEMPOL 4-hydroxy-2,2,6,6-tetramethylpiperidinyloxy, free radical

ROS reactive oxygen species

## Competing interests

The author(s) declare that they have no competing interests.

## Authors' contributions

D.L. optimized and performed immunoblotting and PCR, M.H. developed and performed PCR, A.P. developed the NBT staining procedure and photographed fluorescent and Varel images, J.F. and I.S. formulated experimental design and methodologies, I.S. performed cell culture, immunoblotting and wrote the manuscript.
